# Supplementary material for: A nanobody-based fluorescent reporter reveals human α-synuclein in the cell cytosol
Source: Nat Commun. 2020 Jun 1;11:2729. doi: 10.1038/s41467-020-16575-0 (PMC7264335; doi:10.1038/s41467-020-16575-0)
Supplement: Supplementary file 1 — Supplementary Information [file 41467_2020_16575_MOESM1_ESM.pdf]

Supplementary Information

A nanobody-based fluorescent reporter reveals human α-synuclein in the cell cytosol

Christoph Gerdes[1], Natalia Waal[2], Thomas Offner[3,4], Eugenio F. Fornasiero[1], Nora Wender[1], Hannes Verbarg[1], Ivan Manzini[3,4], Claudia Trenkwalder[5,6], Brit Mollenhauer[6,7], Timo Strohäker[8], Markus Zweckstetter[8,9], Stefan Becker[9], Silvio O. Rizzoli[1], Fitnat Buket Basmanav[1,10,§], Felipe Opazo[1,2,§,*]

[1]Department of Neuro- and Sensory Physiology, University Medical Center Göttingen, D-37073 Göttingen, Germany

[2]Center for Biostructural Imaging of Neurodegeneration (BIN), University Medical Center Göttingen, D-37073 Göttingen, Germany

[3]Institute of Animal Physiology, Department of Animal Physiology and Molecular Biomedicine, Justus-Liebig University Giessen, 35390 Giessen, Germany.

[4]Institute of Neurophysiology and Cellular Biophysics, University of Göttingen, Göttingen, Germany

[5]Department of Neurosurgery, University Medical Center Göttingen, D-37075 Göttingen, Germany

[6]Paracelsus-Elena-Klinik, Klinikstraße 16, 34128 Kassel, Germany

[7]Department of Neurology, University Medical Center Göttingen, D-37075 Göttingen, Germany

[8]German Center for Neurodegenerative Diseases (DZNE), Von-Siebold-Str. 3a, 37075 Göttingen, Germany

[9]Department for NMR-based Structural Biology, Max Planck Institute for Biophysical Chemistry, Am Faßberg 11, 37077 Göttingen, Germany

[10]Campus Laboratory for Advanced Imaging, Microscopy and Spectroscopy, University of Göttingen, D-37073 Göttingen, Germany

[§] These authors contributed equally

*Correspondence should be addressed to:

Felipe Opazo (fopazo@gwdg.de)

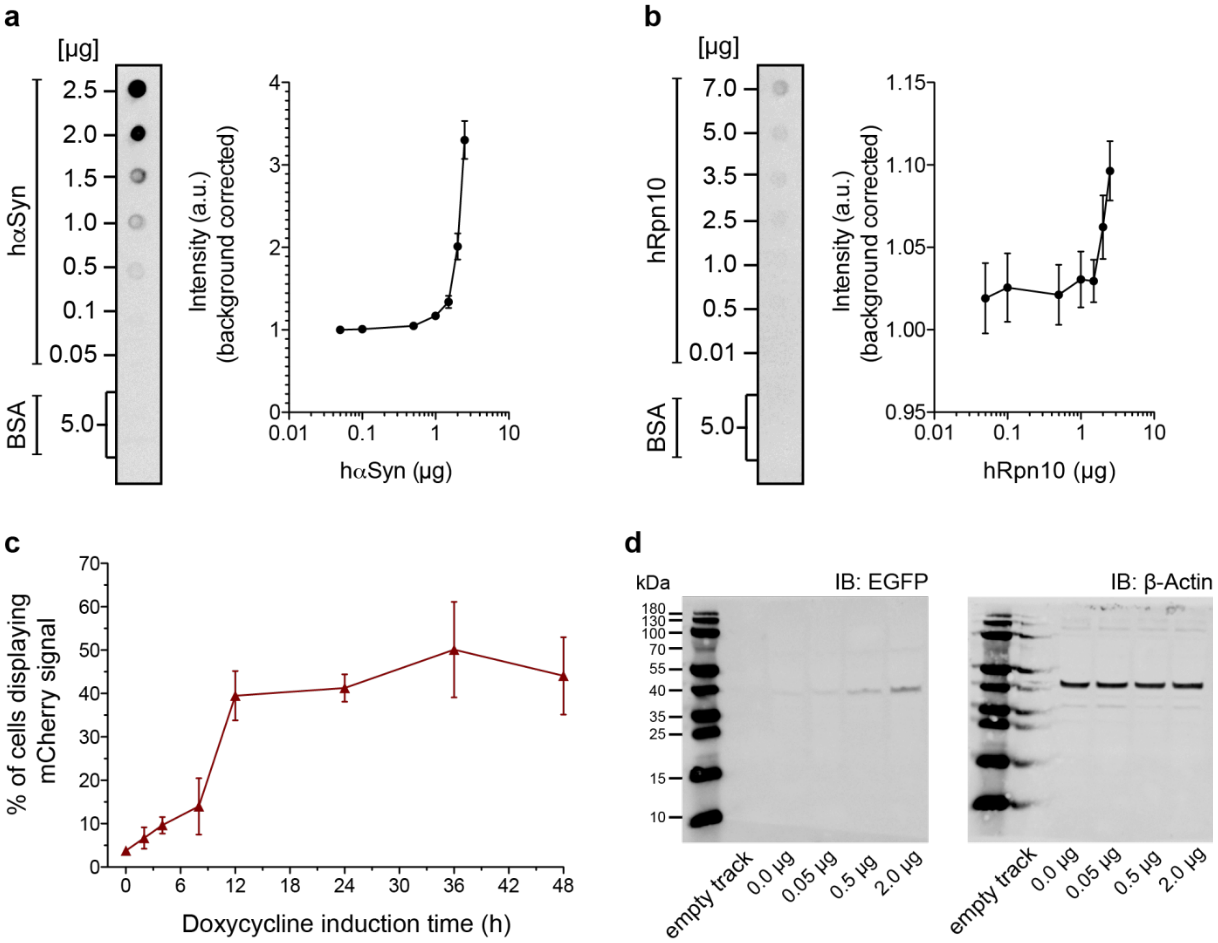

Supplementary Figure 1 | NbSyn87 binding to hαSyn and hRpn10 and the biochemical characterization of Reporter-cells. (**a, b**) Increasing amounts of hαSyn (**a**) or hRpn10 (**b**) were spotted on a nitrocellulose membrane and detected using NbSyn87 directly coupled to Alexa 647. Bovine serum albumin (BSA) was spotted as control protein and its signal was used as background for normalization. Error bars represent the SEM from 3 independent experiments. (**c**) Induction response curve of the Reporter-cells to determine the optimal duration of doxycycline administration at a concentration of 0.5 µg/ml. Error bars represent the SEM from 4 independent experiments. (**d**) Full Western blots membranes from Fig. 1f.

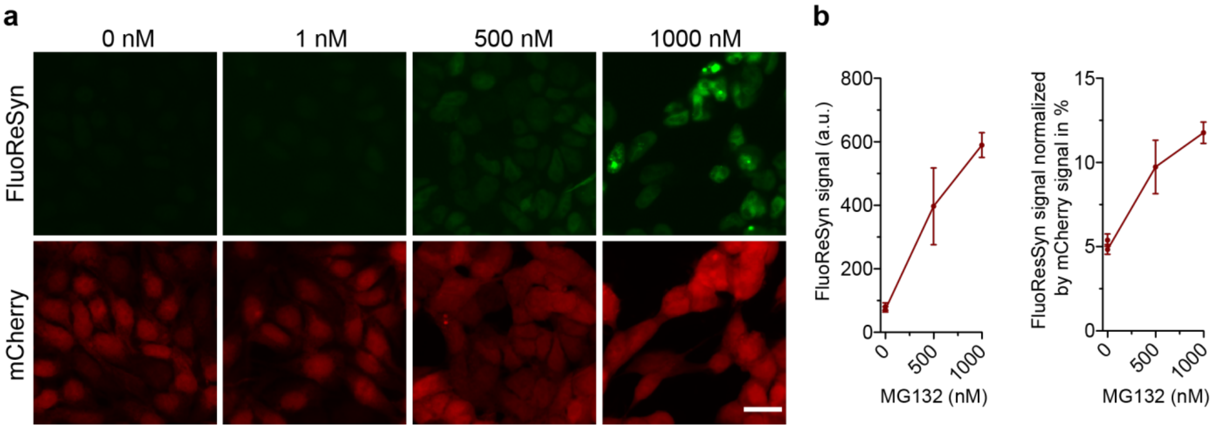

Supplementary Figure 2 | Determination of the optimal concentration for MG132 administration. (**a**) Fully induced Reporter-cells were exposed to different concentrations of MG132 for 16h. The mCherry signal (red) indicate that the cells are producing FluoReSyn, however, only starting from 500 nM of MG132, the FluoReSyn signal begins to appear (green). (**b**) Quantification of the FluoReSyn signal (green) in arbitrary units (a.u.) or normalized to the signal of mCherry. Error bars represent the SEM from 3 independent experiments with several hundreds of cells analyzed per experiment.

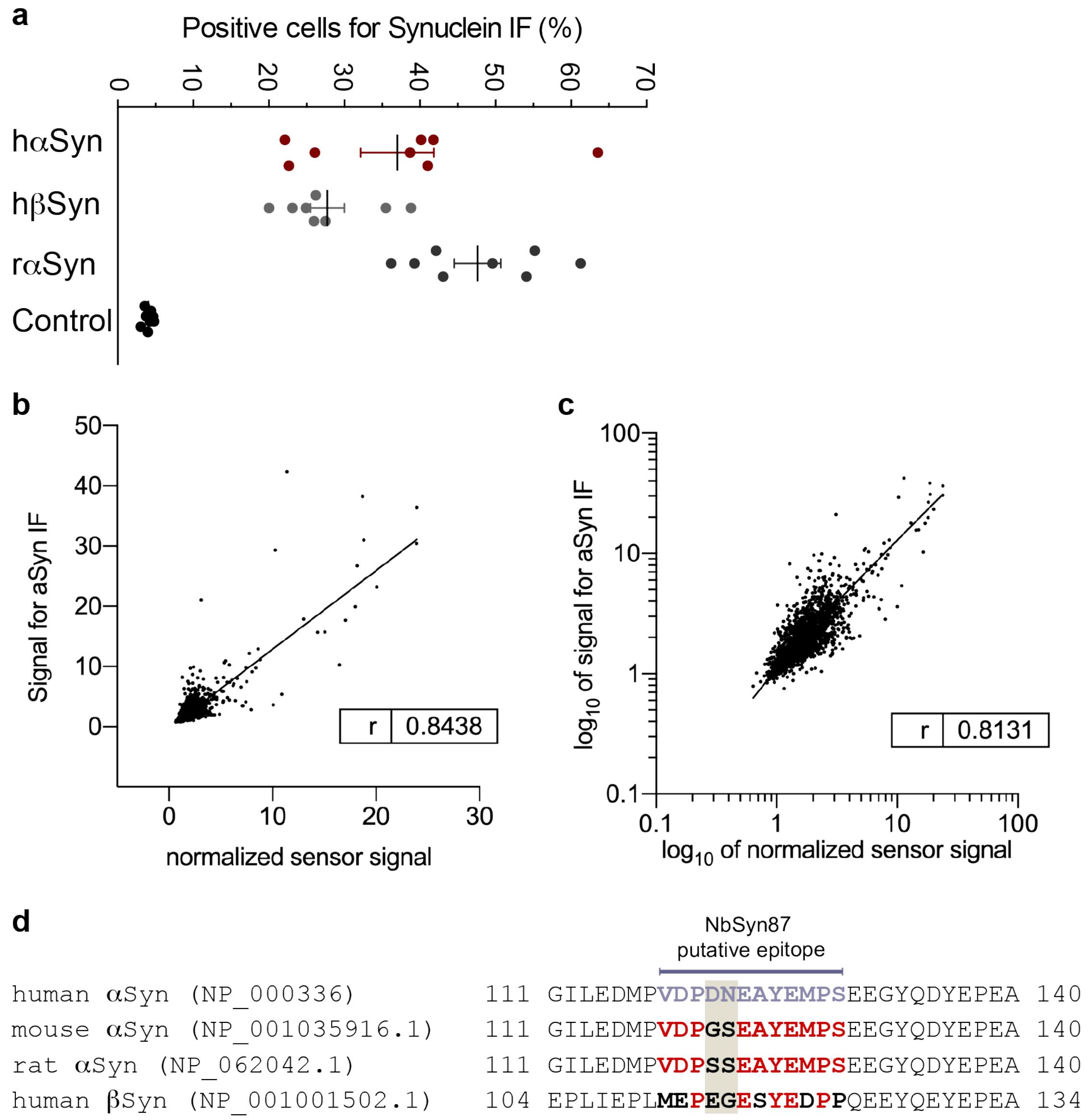

Supplementary Figure 3 | Specificity of FluoReSyn for hαSyn. (**a**) Quantification of the signal obtained after immunostaining synucleins with a pan-Synuclein antibody. Lines represent the mean and the error bars the SEM from n= 8. (**b**) Pearson´s correlation analysis between the signal from FluoReSyn and immunostaining of αSyn. Signals from αSyn immunofluorescence (IF) and FluoReSyn were obtained by analyzing regions of interest (ROI) determined automatically. The ROIs were obtained by identifying all cell nuclei in the DAPI channel, followed by a morphological dilation of the nucleus ROIs, to account for the rest of the cell surfaces. (**b**) Raw values, in arbitrary units. (**c**) Double logarithmic graph of the same values. The Pearson´s correlation coefficient (r) was 0.8438 for (b) with a two-tailed p <0.0001 (****), and r = 0.8131 for (c) with a two-tailed p <0.0001(****). (**d**) Amino acid sequence alignment of the putative epitope recognized by NbSyn87 for hαSyn (letters in light purple), mouse and rat αSyn or human βSyn. (accession numbers for each sequence are on the figure). Red letters show a match amino acid to the putative epitope, black letters represent mismatches. The core difference between the sequences is highlighted by a background box.

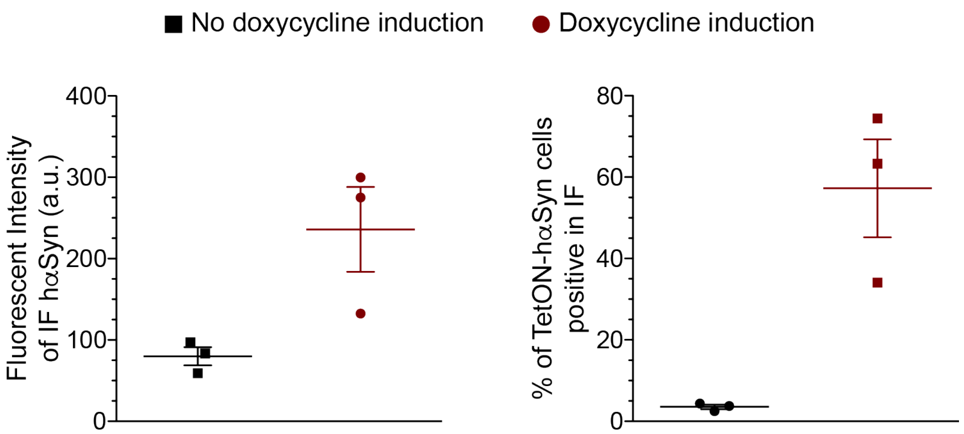

Supplementary Figure 4 | TetON induction of stably-transfected HEK293 cells expressing untagged hαSyn. (left plot) Fluorescence intensity of cells immunoassayed for synuclein after being induced or not with 0.5 µg/ml of doxycycline for 16h. (right plot) Percentage of cells displaying a positive immunofluorescense (IF) signal for synuclein. Error bars represent the SEM from 3 independent experiments (n= 3).

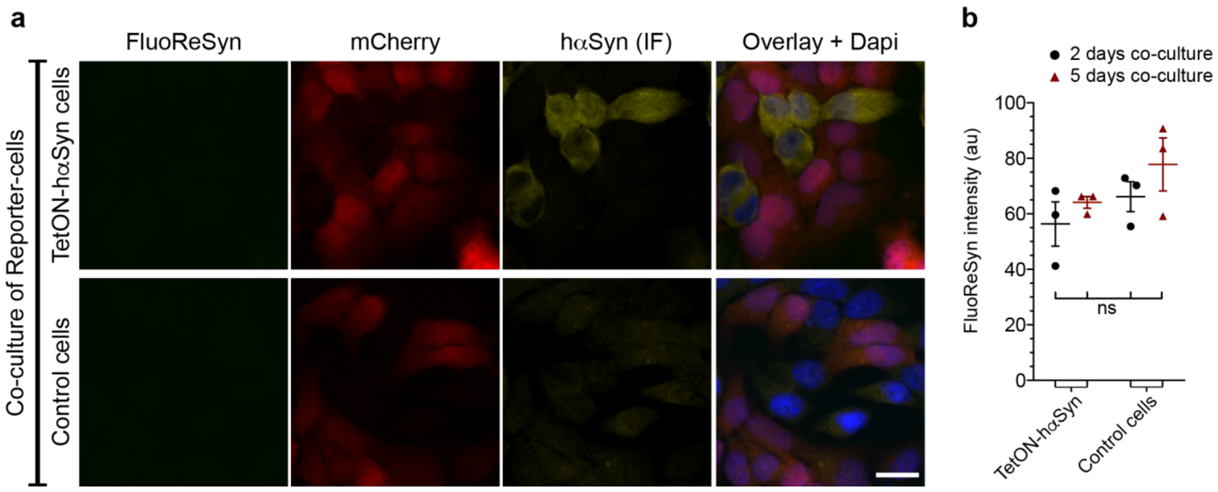

Supplementary Figure 5 | Co-culture of Reporter cells with TetON-hαSyn cells. (**a**) Reporter-cells co-cultured with either a doxycycline inducible hαSyn expressing cell line (TetON-hαSyn) or the wildtype HEK293 cell line without endogenous hαSyn expression. Induced Reporter-cells can be identified by their mCherry signal while induced TetON-hαSyn cells can be identified by the hαSyn immunofluorescence (IF, yellow). (**b**) Quantitative analysis of FluoReSyn signal intensity of cells with mCherry above 300 arbitrary units (a.u.) after 2 or 5 days of co-culturing. Scatter plots show 3 independent experiments (n= 3) ± SEM for all condition. Significance was assessed by One-Way ANOVA and Tukey's Post-hoc test. ns, non-significant. Per replication and condition more than 550 cells were analyzed.

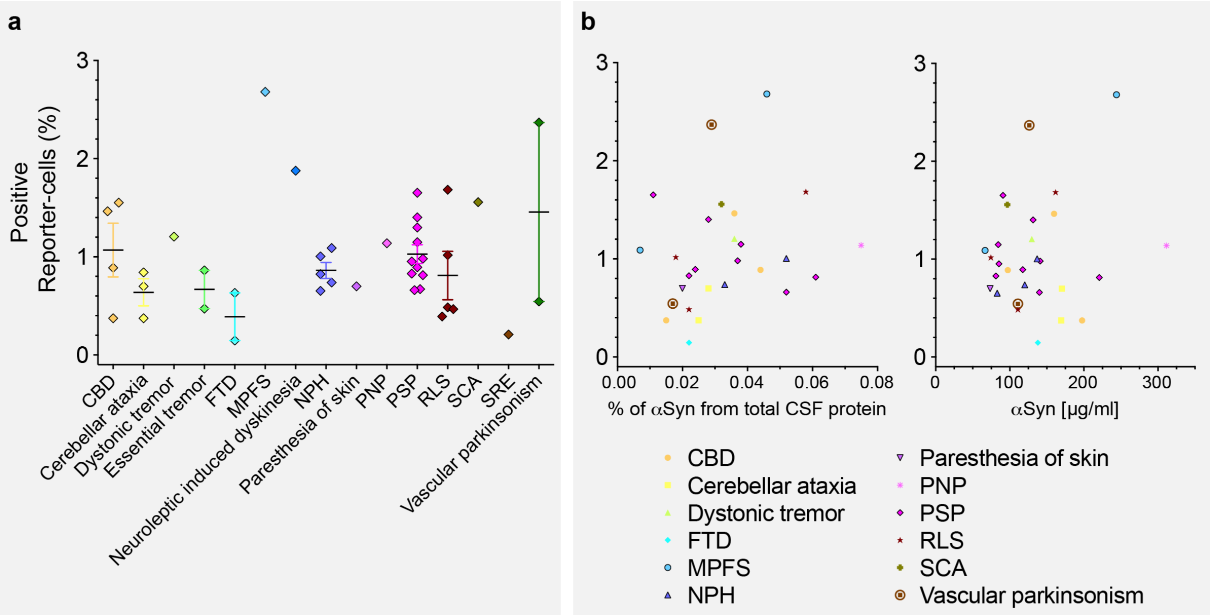

Supplementary Figure 6 | Reporter cell line signals plotted according to the clinical category of the patients and to their hαSyn content in CSF. (**a**) The same data as plotted in Fig 7b, displayed according to the patient clinical description. Note that some categories have only one patient. Each patient is represented as a data point (n), the mean values are depicted with a horizontal lines and error bars represent the SEM for each category. (**b**) The percentage of positive Reporter cells, plotted against (left) the mass percentage of hαSyn (within all CSF proteins) or (right) the concentration of hαSyn present in the CSF (µg/ml). Note that the concentrations of hαSyn or the total protein in CSF were not available for some cases, and are therefore missing in the graphs. CBD, corticobasal dementia; MPFS, muscular pain-fasciculation syndrome; FTD, frontotemporal dementia; NPH, normal pressure hydrocephalus; PNP, peripheral neuropathy; PSP, progressive supranuclear palsy; RLS, restless legs syndrome; SCA, spinocerebellar ataxia; SRE, steroid responsive encephalopathy. Pearson´s correlation (r) was 0.6778 when hαSyn concentrations were normalized by total protein in the CSF (two-tailed p = 0.5260, not significant), and r = -0.3424 when using the direct hαSyn concentrations (two-tailed p =0.7775, not-significant).

Supplementary Table 1. Demographic and clinical characteristics of the individuals in the CSF study

ID	Gender	Age	Diagnosis
1	f	65	Muscular pain-fasciculation syndrome (MPFS)
2	f	73	RLS
3	m	77	FTD
4	f	60	RLS
5	m	73	CBD
6	m	74	Cerebellar ataxia
7	m	68	PSP
8	f	82	PSP
9	f	71	PSP
10	f	64	FTD
11	m	87	Vascular parkinsonism
12	m	88	Essential tremor
13	m	76	Vascular parkinsonism
14	f	81	PSP
15	m	70	PSP
16	f	81	NPH without evidence for other neurodegenerative disorders
17	f	73	CBD
18	m	76	RLS
19	m	73	NPH without evidence for other neurodegenerative disorders
20	f	69	NPH without evidence for other neurodegenerative disorders
21	f	77	NPH without evidence for other neurodegenerative disorders
22	m	75	PSP
23	f	73	CBD
24	f	64	Cerebellar ataxia
25	m	60	PSP
26	f	75	SCA
27	f	77	Steroid responsive encephalopathy associated with athyroiditis
28	f	71	Essential tremor
29	m	69	PSP
30	f	75	Dystonic tremor
31	m	84	PNP
32	f	35	Paraesthesia of skin
33	f	65	Neuroleptic-induced dyskinesia
34	m	48	RLS
35	f	59	PSP
36	m	76	PSP
37	m	65	NPH without evidence for other neurodegenerative disorders
38	m	60	Cerebellar ataxia
39	f	71	CBD
40	f	80	Benign paroxysmal positional vertigo
41	f	73	RLS
42	m	67	PSP

m, male; f, female; CBD, corticobasal dementia; FTD, frontotemporal dementia; NPH, normal pressure hydrocephalus; PNP, peripheral neuropathy; PSP, progressive supranuclear palsy; RLS, restless legs syndrome; SCA, spinocerebellar ataxia.
