# Supplementary material for: A nanobody-based fluorescent reporter reveals human α-synuclein in the cell cytosol
Source: Nat Commun. 2020 Jun 1;11:2729. doi: 10.1038/s41467-020-16575-0 (PMC7264335; doi:10.1038/s41467-020-16575-0)
Supplement: Supplementary file 3 — Reporting Summary [file 41467_2020_16575_MOESM3_ESM.pdf]

nature research

Corresponding author(s): Felipe Opazo
Last updated by author(s): Apr 23, 2020

Reporting Summary

Nature Research wishes to improve the reproducibility of the work that we publish. This form provides structure for consistency and transparency in reporting. For further information on Nature Research policies, see Authors & Referees and the Editorial Policy Checklist.

Statistics

For all statistical analyses, confirm that the following items are present in the figure legend, table legend, main text, or Methods section.

n/a	Confirmed	
☐	☒	The exact sample size (*n*) for each experimental group/condition, given as a discrete number and unit of measurement
☐	☒	A statement on whether measurements were taken from distinct samples or whether the same sample was measured repeatedly
☐	☒	The statistical test(s) used AND whether they are one- or two-sided<br>*Only common tests should be described solely by name; describe more complex techniques in the Methods section.*
☒	☐	A description of all covariates tested
☐	☒	A description of any assumptions or corrections, such as tests of normality and adjustment for multiple comparisons
☐	☒	A full description of the statistical parameters including central tendency (e.g. means) or other basic estimates (e.g. regression coefficient) AND variation (e.g. standard deviation) or associated estimates of uncertainty (e.g. confidence intervals)
☐	☒	For null hypothesis testing, the test statistic (e.g. *F*, *t*, *r*) with confidence intervals, effect sizes, degrees of freedom and *P* value noted<br>*Give P values as exact values whenever suitable.*
☒	☐	For Bayesian analysis, information on the choice of priors and Markov chain Monte Carlo settings
☒	☐	For hierarchical and complex designs, identification of the appropriate level for tests and full reporting of outcomes
☐	☒	Estimates of effect sizes (e.g. Cohen's *d*, Pearson's *r*), indicating how they were calculated

Our web collection on statistics for biologists contains articles on many of the points above.

Software and code

Policy information about availability of computer code

Data collection	Epifluorescence microscopy: Olympus IX71 microscope equipped with a CellSens Dimension 2.3 Software<br>Nikon Eclipse microscope<br>Nikon A1R MP (2 photon)<br>Western Blot imaging: LiCor Sytem Odyssey Clx<br>ÄKTA chromatography: UNICORN™ 7.0.2 software (GE Healthcare Life Sciences)
Data analysis	GraphPad Prism 5, 2012 GraphPad Software Inc.;<br>Matlab Version 2014b, 2007, Mathworks Inc;<br>Sigma Plot 11 (Systat Software, San Jose, CA, USA);<br>ImageJ, Fiji, version: 2.0.0-rc-69/1.52p; Build 269a0ad53f<br>Phyton Seaborn package, python.org; version 0.9; 10.5281/zenodo.1313201

For manuscripts utilizing custom algorithms or software that are central to the research but not yet described in published literature, software must be made available to editors/reviewers. We strongly encourage code deposition in a community repository (e.g. GitHub). See the Nature Research guidelines for submitting code & software for further information.

Data

Policy information about availability of data

All manuscripts must include a data availability statement. This statement should provide the following information, where applicable:

- Accession codes, unique identifiers, or web links for publicly available datasets
- A list of figures that have associated raw data
- A description of any restrictions on data availability

Data Availability
The data that supports the finding of this study is readily available within this paper, its Supplementary file and in the Source Data file. The data set for Figure 1e, 2b,

2c, 3i, 4b, 5b, 6b, 6c, 7b and Supplementary Figures 1a, 1b, 1c, 3a, 3b, 5b and 6 are provided in the Source Data file. All data sets generated during and/or analyzed during the current study are available from the corresponding author on reasonable request.

Field-specific reporting

Please select the one below that is the best fit for your research. If you are not sure, read the appropriate sections before making your selection.

☒ Life sciences ☐ Behavioural & social sciences ☐ Ecological, evolutionary & environmental sciences

For a reference copy of the document with all sections, see nature.com/documents/nr-reporting-summary-flat.pdf

Life sciences study design

All studies must disclose on these points even when the disclosure is negative.

Sample size	Sample size calculation were not performed a priori. In quantitative data, the aim was to collect as much data as possible, the sample size provided a sufficient accuracy to distinguish statistically significant differences between conditions. In descriptive experiments we chose a sample size that was large enough to show difference between the conditions.
Data exclusions	No data were excluded from the analysis, except raw images which were out of focus.
Replication	Replicates and independent experiments were carefully kept that way. Most of our Experiments were replicated independently at least 3 times. Specific n can be found in the each Figure legend.
Randomization	Randomization was not performed for this study.
Blinding	Blinding was not performed in this study.

Reporting for specific materials, systems and methods

We require information from authors about some types of materials, experimental systems and methods used in many studies. Here, indicate whether each material, system or method listed is relevant to your study. If you are not sure if a list item applies to your research, read the appropriate section before selecting a response.

Materials & experimental systems

n/a	Involved in the study	
☐	☒	Antibodies
☐	☒	Eukaryotic cell lines
☒	☐	Palaeontology
☐	☒	Animals and other organisms
☐	☒	Human research participants
☒	☐	Clinical data

Methods

n/a	Involved in the study	
☒	☐	ChIP-seq
☒	☐	Flow cytometry
☒	☐	MRI-based neuroimaging

Antibodies

Antibodies used	rabbit polyclonal anti-α/β-Synuclein antibody; dilution used 1:500; Cat. No. 128002; Synaptic System GmbH<br>guinea pig polyclonal anti-synaptophysin antibody; dilution 1:100; Cat. No. 101004; Synaptic System GmbH<br>donkey anti-guinea pig labeled with ATTO 647N; dilution 1:500; Cat. No. N0602-At647N-S; Synaptic System GmbH<br>donkey anti-rabbit; dilution 1:500; Cat. No. 711-175-152, Dianova GmbH<br>mouse monoclonal (clone 3E6) anti EGFP; dilution 1:500; Cat. No. A11120, Thermo-Scientific<br>rabbit polyclonal (affinity purified) anti beta-Actin directly labeled with Cy5; dilution 1:1000; Cat. No. 251003, Synaptic System GmbH
Validation	anti-α/β-Synuclein: antiserum, Synthetic peptide corresponding to AA 2 to 25 from human α-Synuclein; Reacts with: rat, mouse, zebrafish and human α synuclein and β synuclein. Validated for IF, IHC.<br>anti-synaptophysin antibody:Synthetic peptide corresponding to AA 301 to 313 from human Synaptophysin1,<br>Reacts with: human, rat, mouse, hamster, cow, chicken, frog. Validated fro IF and IHC.<br>Specific for synaptophysin 1, no cross-reactivity to other synaptophysins.<br>Mouse monoclonal anti EGFP (clone 3E6) validated in IF, IHC, WB and more applications source Thermo-fisher.<br>Rabit polyclonal anti Beta actin, antigen is the AA2 to AA16 of mouse Beta Actin, Externally validated for WB stated in suppliers webpage: Synaptic System GmbH

Eukaryotic cell lines

Policy information about cell lines

Cell line source(s)	Wildtype HEK293 were purchased from the German Collection for Microorganisms and Cell Cultures (DSMZ), Braunschweig, Germany. Stably transfected lines like the Report cell line and the aSyn cell line were produced by Sirion Biotech GmbH (Martinsried, Germany). A full report on their generation was provided using DZMZ HEK-203 cell lines.
Authentication	We relied on the authentication from DSMZ and Sirion Biotechnology GmbH
Mycoplasma contamination	Cells were tested negative for mycoplasma.
Commonly misidentified lines (See ICLAC register)	HEK-293 were not reported by the ICLAC to be misidentified.

Animals and other organisms

Policy information about studies involving animals; ARRIVE guidelines recommended for reporting animal research

Laboratory animals	Xenopus laevis tadpoles (albinos)
Wild animals	*Provide details on animals observed in or captured in the field; report species, sex and age where possible. Describe how animals were caught and transported and what happened to captive animals after the study (if killed, explain why and describe method; if released, say where and when) OR state that the study did not involve wild animals.*
Field-collected samples	*For laboratory work with field-collected samples, describe all relevant parameters such as housing, maintenance, temperature, photoperiod and end-of-experiment protocol OR state that the study did not involve samples collected from the field.*
Ethics oversight	All procedures for animal handling were approved by the governmental animal care and use office (Niedersächsisches Landesamt für Verbraucherschutz und Lebensmittelsicherheit, Oldenburg, Germany, Az.12/0779) and were in accordance with the German Animal Welfare Act as well as with the guidelines of the Göttingen University Committee for Ethics in Animal Experimentation

Note that full information on the approval of the study protocol must also be provided in the manuscript.

Human research participants

Policy information about studies involving human research participants

Population characteristics	Study participants consisted of individuals who were in treatment at the Paracelsus Elena Klinik, Kassel, Germany and had been diagnosed with a variety of neurological disorders non-related to αSyn aggregation disorders.
Recruitment	CSF samples from all individuals were collected after the informed consent of the participant at the Paracelsus Elena Klinik (Kassel, Germany) in accordance with the principles of Declaration of Helsinki and following identical standard operating procedures.
Ethics oversight	The use of the CSF samples in this study was approved by the ethical committee of the Medical Center Göttingen with the approval numbers 36/7/02 and 9/7/04

Note that full information on the approval of the study protocol must also be provided in the manuscript.
